# *Mycobacterium ahvazicum* sp. nov., the nineteenth species of the *Mycobacterium simiae* complex

**DOI:** 10.1038/s41598-018-22526-z

**Published:** 2018-03-07

**Authors:** Amar Bouam, Parvin Heidarieh, Abodolrazagh Hashemi Shahraki, Fazel Pourahmad, Mehdi Mirsaeidi, Mohamad Hashemzadeh, Emeline Baptiste, Nicholas Armstrong, Anthony Levasseur, Catherine Robert, Michel Drancourt

**Affiliations:** 1 Unité des Rickettsies, CNRS UMR 7278 Faculté de Médecine, Aix-Marseille-Université, Marseille, France; 2 MEPHI, Aix Marseille Université, IRD, IHU Méditerranée Infection, Marseille, France; 3 Department of Microbiology, School of Medicine, Alborz University of Medical Science, Alborz, Iran; 4 Department of Epidemiology, Pasteur Institute of Iran, Tehran, Iran; 5 School of Veterinary Medicine, Ilam University, Ilam, Iran; 6 Division of Pulmonary and Critical Care, University of Miami, Miami, FL USA; 7 Health Institute, Infectious and Tropical Disease Research Center, Ahvaz Jundishapur University of Medical Sciences, Ahvaz, Iran; 8 VITROME, Aix Marseille Université, IRD, IHU Méditerranée Infection, Marseille, France

## Abstract

Four slowly growing mycobacteria isolates were isolated from the respiratory tract and soft tissue biopsies collected in four unrelated patients in Iran. Conventional phenotypic tests indicated that these four isolates were identical to *Mycobacterium lentiflavum* while 16S rRNA gene sequencing yielded a unique sequence separated from that of *M*. *lentiflavum*. One representative strain AFP-003^T^ was characterized as comprising a 6,121,237-bp chromosome (66.24% guanosine-cytosine content) encoding for 5,758 protein-coding genes, 50 tRNA and one complete rRNA operon. A total of 2,876 proteins were found to be associated with the mobilome, including 195 phage proteins. A total of 1,235 proteins were found to be associated with virulence and 96 with toxin/antitoxin systems. The genome of AFP-003^T^ has the genetic potential to produce secondary metabolites, with 39 genes found to be associated with polyketide synthases and non-ribosomal peptide syntases and 11 genes encoding for bacteriocins. Two regions encoding putative prophages and three OriC regions separated by the dnaA gene were predicted. Strain AFP-003^T^ genome exhibits 86% average nucleotide identity with *Mycobacterium genavense* genome. Genetic and genomic data indicate that strain AFP-003^T^ is representative of a novel *Mycobacterium* species that we named *Mycobacterium ahvazicum*, the nineteenth species of the expanding *Mycobacterium simiae* complex.

## Introduction

Investigating non-tuberculous mycobacteria in Iran recently succeeded in the characterization of two new species of rapidly growing scotochromogenic mycobacteria, i.e. *Mycobacterium iranicum* isolated in 2009 from the bronchoalveolar lavage of a 60-year-old female patient suffering from chronic pulmonary disease^1^ and *Mycobacterium celeriflavum* isolated in 2010 from the sputum of a 44-year-old male suffering from chronic obstructive pulmonary disease^2^. We recently had the opportunity to investigate four clinical isolates made in Iran and we proved they were representative of one additional new species of non-tuberculous *Mycobacterium* that we named *Mycobacterium ahvazicum*. AFP-003^T^ strain was isolated in 2009 from the sputum and bronchoalveolar lavage specimen of a 68-year-old Iranian female suffering from chronic pulmonary disease. Phenotypic and genetic investigations based on 16S rRNA and *rpo*B gene sequencing revealed that the AFP-003 strain was probably representative of a new species of non-tuberculous *Mycobacterium* in Iran. Following the isolation of strain AFP-003, three other strains exhibiting the very same phenotypic and genetic characters were isolated in Iran: strain AFP-004 was isolated in 2009 from a biopsy of diseased soft tissues in a 49-year-HIV-infected patient, strain MH1 was isolated in 2013 from sputum in a 60-year-old male patient and strain RW4 was isolated in 2015 from a bronchoalveolar lavage specimen in a 19-year-old patient suffering from asthma. Strain AFP-003 was then fully characterized as a type strain and then designated as strain AFP-003^T^.

## Results

**Figure 1:**
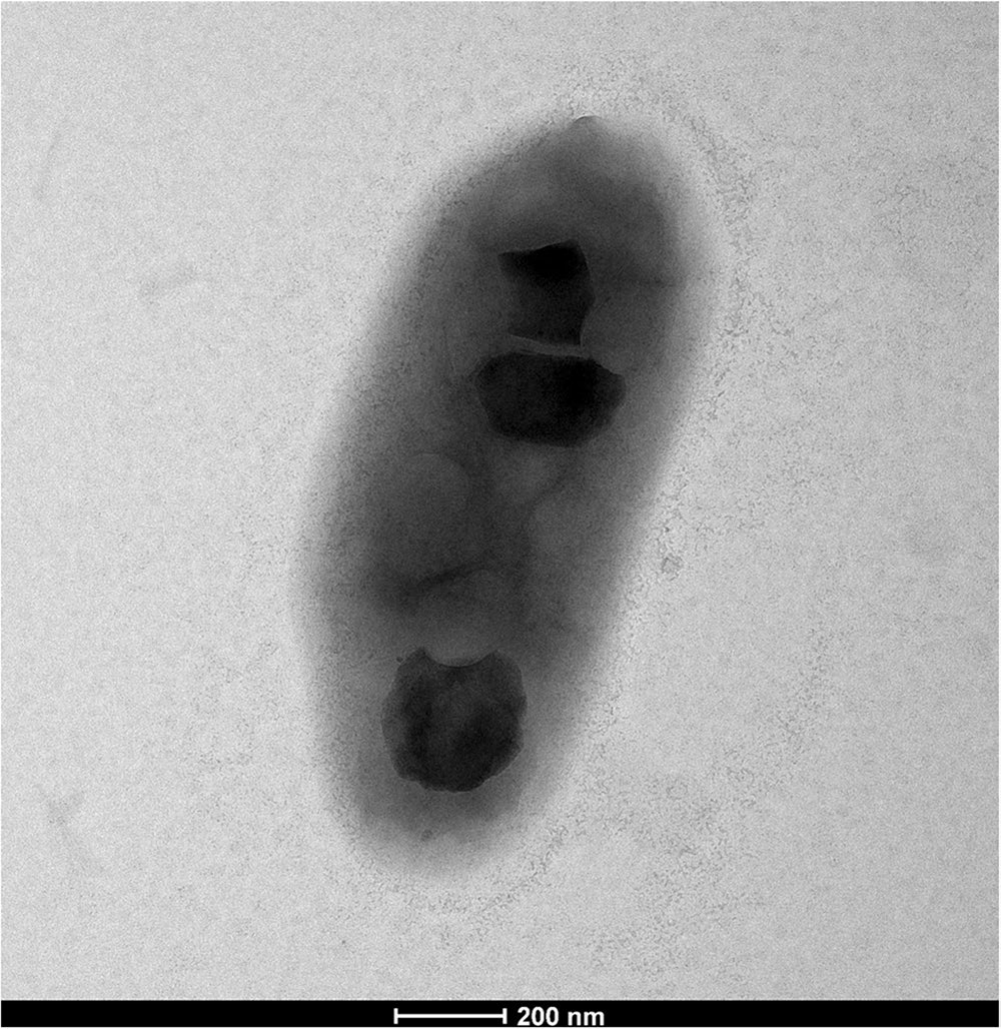
Transmission electron microscopy of *Mycobacterium ahvazicum* strain AFP-003^T^. The scale bar represents 200 nm.

AFP-003^T^ yielded smooth, yellow and scotochromogenic colonies after 3–4-weeks of incubation on Löwenstein-Jensen medium at a temperature between 33 °C and 42 °C, with an optimal growth at 37 °C; but it did not grow on Löwenstein-Jensen containing 5% NaCl. The observation of colonies by electron microscopy showed rod-shaped bacilli measuring 1.53 ± 0.32 µm long and 0.64 ± 0.07 µm large (Fig. 1). AFP-003^T^ exhibited a heat-stable (68 °C) catalase but was negative for semi-quantitative catalase; and negative for urease activity, iron uptake, tellurite reduction, arylsulfatase activity after three days, niacin production, nitrate reduction, Tween hydrolysis and growth on MacConkey agar without crystal violet. These conventional phenotypic tests were not sufficient to differentiate AFP-003^T^ from *Mycobacterium lentiflavum* (Table 1). However, AFP-003^T^ reproducible matrix-assisted laser desorption ionization-time of flight-mass spectrometry (MALDI-TOF-MS) profile did not match any of the profiles entered in the Bruker database (version December, 2015, including *M*. *lentiflavum*), suggesting that AFP-003^T^ was not identifiable as *M*. *lentiflavum* and could indeed be representative of a hitherto undescribed species of *Mycobacterium*.

**Table 1:** Phenotypic characteristics of *M*. *ahvazicum* strain AFP-003^T^ and related slowly growing mycobacteria species.

Characteristics	1	2	3	4	5
Growth at:					
30 °C	−	+	+	+	+
37 °C	+	+	+	+	−
42 °C	+	−	+	+	−
Pigmentation	S/yellow	S/yellow	SP/yellow	N	N
Arysulfatase	−	−	+	+	+
Catalase (68 °C)	+	+	+	+	+
Catalase (>45 mm of foam)	−	−	+	−	−
Nitrate reduction	−	−	−	+	−
Tween 80 hydrolysis	−	−	−	−	−
Niacin production	−	−	V	−	−
Tellurite reduction	−	−	+	ND	+
Urease activity	−	−	+	+	−
Tolerance to NaCl (5%, w/v)	−	−	−	−	−
Growth on MacConkey agar without crystal violet	−	−	ND	ND	−

1, *M*. *ahvazicum* sp. nov.; 2, *M*. *lentiflavum*; 3, *M*. *simiae*; 4, *M*. *triplex*; 5, *M*. *stomatepiae*. S; scotochromogenic, SP; scotochromogenic or photochromogenic, N; nonchromogenic, ND; Not determined; −, negative; +, positive; V, variable. Data for species other than *M*. *ahvazicum* sp. nov are from Levi *et al*.^22^, Pourahmad *et al*.^23^ and Tortoli (2006).

**Figure 2:**
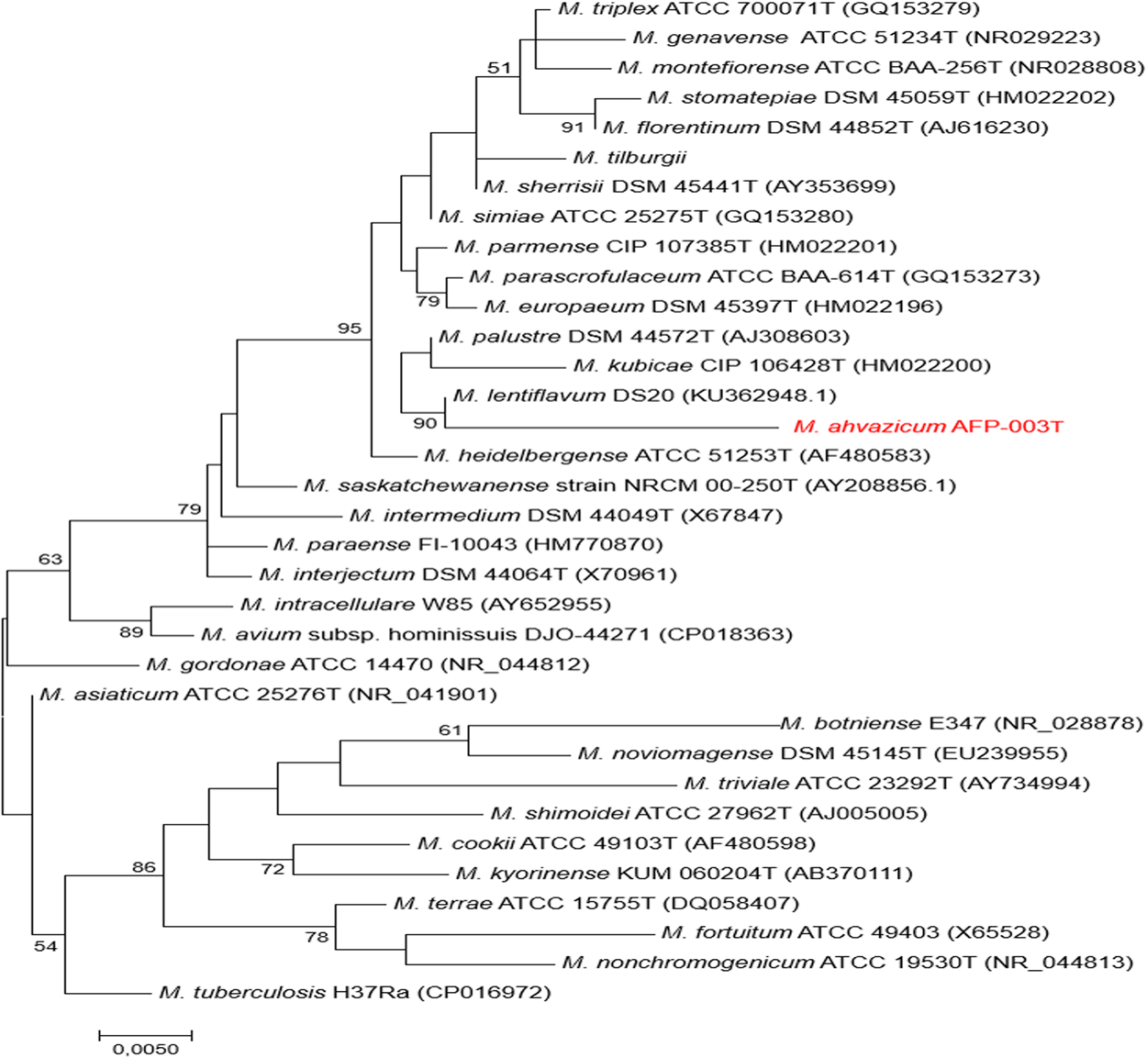
Phylogenetic tree based on the 16S rRNA gene sequence indicating the phylogenetic position of *M*. *ahvazicum* strain AFP-003^T^ relative to other species of *M*. *simiae* and other mycobacteria species including *Mycobacterium tuberculosis* as an out group. Sequences were aligned using CLUSTLE W implemented on MEGA7^33^. The analysis involved 34 nucleotide sequences. All positions containing gaps and missing data were eliminated. There were a total of 1,233 positions in the final dataset. Phylogenetic inferences obtained using the maximum likelihood method based on the Tamura and Nei model (bootstrapped 1000 times). Bootstrap values >50% are given at nodes. Bar, 0.005 substitutions per nucleotide position.

AFP-003^T^ was then shown to be *in vitro* susceptible to ciprofloxacin, clarithromycin and rifampicin (Table 2). Furthermore, Biolog® Phenotype MicroArray test showed that AFP-003^T^ grew under other 14 inhibitory chemical conditions including minocycline, lincomycin, guanidine HCl, Niaproof Anionic Surfactant, vancomycin, tetrazolium violet, tetrazolium blue, nalidixic acid, lithium chloride, potassium tellurite, aztreonam, sodium butyrate and sodium bromate; and was able to metabolize eight carbon sources including α D-glucose, glucuronamide, methyl pyruvate, α-keto-glutaric, α-keto-butyric acid, acetoacetic acid, propionic acid and acetic acid (Table 3). The 16S rRNA gene sequence’s (GenBank accession: LT797535) highest similarity was of 98.1%, 97.8%, 97.5% and 97.4% with *M*. *lentiflavum* ATCC 51985, *Mycobacterium simiae*, *Mycobacterium triplex* and *Mycobacterium sherrisii*, respectively. Partial *rpo*B gene sequencing was previously shown to be a useful marker to delineate new *Mycobacterium* species^3^ and we sequenced a 619-bp rpoB gene fragment in AFP-003^T^ strain (GenBank accession: FR695853). This sequence’s highest similarity was of 96.43%, 95.55% and 94.95% with *Mycobacterium florentinum* DSM 44852, *Mycobacterium stomatepiae* DSM 45059 and *Mycobacterium genavense* FI-06288 respectively. These values being all below the 97% cut-off value previously proposed to delineate different species among *Mycobacterium*^3^ enforced the suggestion that AFP-003^T^ was representative of a new species belonging to the *M*. *simiae* complex, the largest complex in the genus *Mycobacterium* currently comprising 18 species^4,5^ (Fig. 2).

**Table 2:** Minimum inhibitory concentration of selected antibiotics against two *M*. *ahvazicum* strains.

Drug	MIC range (g /L)		Interpretation*
	AFP-003^T^	AFP-004	
Amikacin	8	32	I
Ciprofloxacin	2	1	S
Clarithromycin	1	4	S
Ethambutol	8	8	R
Rifampicin	0.05	0.05	S
Streptomycin	2	16	I

*I, intermediate; S, susceptible; R, resistant.

**Table 3:** Phenotype Microarray Biolog, Gen III Microplate profile of *M*. *ahvazicum* strain AFP-003^T^.

Position	Substrates	Activity
C 1	alpha-D-Glucose	+
C 12	D-Serine	+
D 12	Minocycline	+
E 10	Lincomycin	+
E 11	Guanidine HCl	+
E 12	Niaproof 4	+
F 6	Glucuronamide	+
F 10	Vancomycin	+
F 11	Tetrazolium Violet	+
F 12	TetrazoliumBlue	+
G 2	Methyl Pyruvate	+
G 6	alpha-Keto-Glutaric Acid	+
G 10	Nalidixic Acid	+
G 11	Lithium Chloride	+
G 12	Potassium Tellurite	+
H 5	alpha-Keto-Butyric Acid	+
H 6	Acetoacetic Acid	+
H 7	Propionic Acid	+
H 8	Acetic Acid	+
H 10	Aztreonam	+
H 11	Sodium Butyrate	+
H 12	Sodium Bromate	+
A 1	Negative Control	
A 2	Dextrin	
A 3	D-Maltose	
A 4	D-Trehalose	
A 5	D-Cellobiose	
A 6	Gentiobiose	
A 7	Sucrose	
A 8	D-Turanose	
A 9	Stachyose	
A 10	D-Raffinose	
A 11	alpha-D-Lactose	
A 12	D-Melibiose	
B 1	beta-Methyl-Dglucoside	
B 2	D-Salicin	
B 3	N-Acetyl-Dglucosamine	
B 4	N-Acetyl-beta-Dmannosamine	
B 5	N-Acetyl-D-galactosamine	
B 6	N-Acetyl Neuraminic Acid	
B 7	D-Mannose	
B 8	D-Fructose	
B 9	D-Galactose	
B 10	1% NaCl	
B 11	4% NaCl	
B 12	8% NaCl	
C 2	3-Methyl Glucose	
C 3	D-Fucose	
C 4	L-Fucose	
C 5	L-Rhamnose	
C 6	Inosine	
C 7	D-Sorbitol	
C 8	D-Mannitol	
C 9	D-Arabitol	
C 10	1% Sodium Lactate	
C 11	Fusidic Acid	
D 1	myo-Inositol	
D 2	Glycerol	
D 3	D-Glucose-6-PO4	
D 4	D-Fructose-6-PO4	
D 5	D-Aspartic Acid	
D 6	D-Serine	
D 7	Gelatin	
D 8	Glycyl-L-Proline	
D 9	L-Alanine	
D 10	Troleandomycin	
D 11	Rifamycin SV	
E 1	L-Arginine	
E 2	L-Aspartic Acid	
E 3	L-Glutamic Acid	
E 4	L-Histidine	
E 5	L-Pyroglutamic Acid	
E 6	L-Serine	
E 7	Pectin	
E 8	D-Galacturonic Acid	
E 9	L-Galactonic Acid Lactone	
F 1	D-Gluconic Acid	
F 2	D-Glucuronic Acid	
F 3	Mucic Acid	
F 4	Quinic Acid	
F 5	D-Saccharic AcidD-Saccharic Acid	
F 7	p-Hydroxy-Phenylacetic Acid	
F 8	D-Lactic Acid Methyl Ester	
F 9	L-Lactic Acid	
G 1	Citric Acid	
G 3	D-Malic Acid	
G 4	L-Malic Acid	
G 5	Bromo-Succinic Acid	
G 7	Tween 40	
G 8	gama-Amino-Butryric Acid	
G 9	alpha-Hydroxy-Butyric Acid	
H 1	β-Hydroxy-D,Lbutyric Acid	
H 2	Formic Acid	
H 3	Positive Control	
H 4	pH 6	
H 9	pH 5

**Figure 3:**
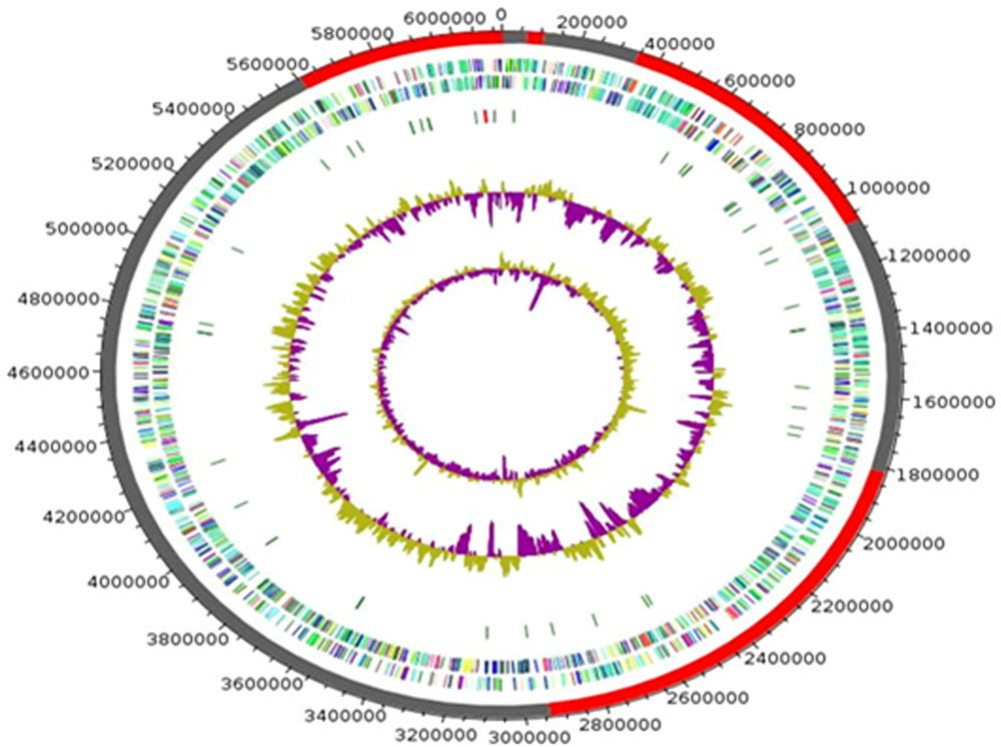
Graphical circular map of the chromosome of *M*. *ahvazicum* strain AFP-003^T^. From outside to the center: Genes on the forward strand colored by COG categories (only genes assigned to COG), genes on the reverse strand colored by COG categories (only gene assigned to COG), RNA genes (tRNAs green, rRNAs red), GC content and GC skew.

**Figure 4:**
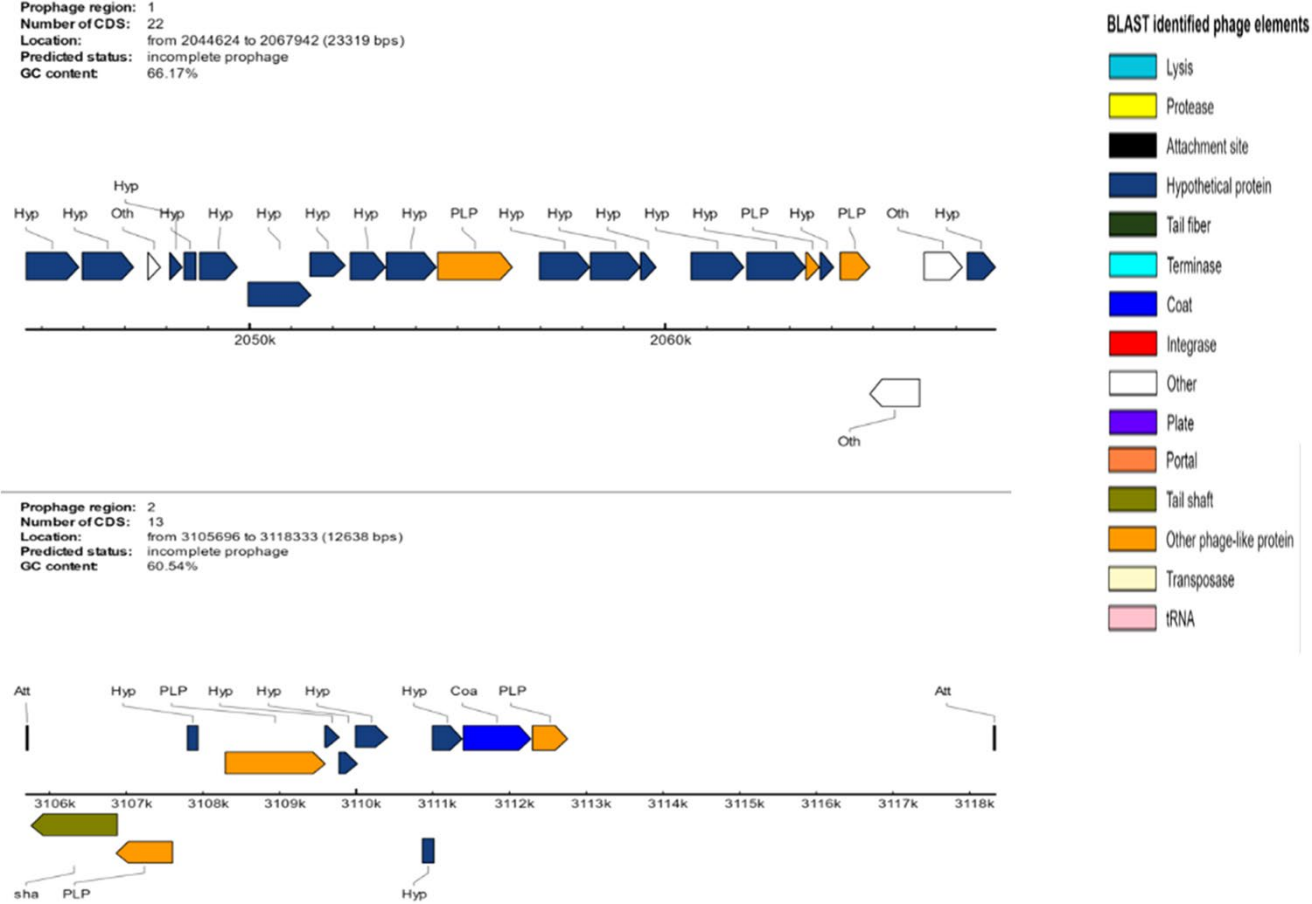
Genomic organization of two uncomplete prophage regions in the genome of *M*. *ahvazicum* strain AFP-003^T^.

We therefore decided to sequence the genome of AFP-003^T^ strain. Genome sequencing yielded four scaffolds indicative of one 6,121,237-bp chromosome (66.24% GC content) without evidence for any extra-chromosomal replicon (Fig. 3). The genome of AFP-003^T^ is smaller than that of *Mycobacterium parascrofulaceum* and *M*. *triplex* (6.564 Mb and 6.383 Mb, respectively) but larger than that of *Mycobacterium interjectum*, *Mycobacterium genavense*, *M*. *sherrisii* and *M*. *simiae* (5.848 Mb, 4.936 Mb, 5.687 Mb and 5.783 Mb, respectively); its GC% content is lower than that of *M*. *parascrofulaceum*, *M*. *interjectum*, *M*. *genavense*, *M*. *sherrisii* and *M*. *triplex* (68.45%, 106 67.91%, 66.92%, 66.92% and 66.6%, respectively) but higher than that of *M*. *simiae* (66.17%). The AFP-003^T^ genome encodes for 5,704 proteins and 52 RNAs including 49 tRNA and one complete rRNA operon in agreement with its classification as slowly growing mycobacterium. A total of 4,869 genes (85.36%) were assigned with putative function (by COGs or by nr blast), whereas 88 genes (1.54%) were identified as ORFans. The remaining genes were annotated as hypothetical proteins without COG assignment (835 genes, 14.64%). A total of 2,617 proteins were found to be associated with the mobilome, including 194 phage proteins. Further genome analysis predicted two incomplete 23.3-Kb and 12.6-Kb prophage regions (Fig. 4). A total of 1,225 proteins were found to be associated with virulence, 95 proteins were associated with toxin/antitoxin systems and 11 genes encoded for bacteriocins while no gene was associated with the resistome. We identified a large number of genes assigned to COG functional categories for transport and metabolism of lipids (10.6%), secondary metabolites biosynthesis, transport and catabolism (6.8%), amino acid transport and metabolism (4.03%) and energy production and conversion (5.3%) (Table 4).

**Table 4:** Number of genes associated in the *M*. *ahvazicum* strain AFP-003^T^ genome with the 25 general COG functional categories. The total % is based on the total number of protein coding genes in the annotated genome.

Code	Value	% of total	Description
[J]	173	3.03	Translation
[A]	1	0.02	Rna processing and modification
[K]	173	3.03	Transcription
[L]	97	1.70	Replication, recombination and repair
[B]	0	0.00	Chromatin structure and dynamics
[D]	29	0.51	Cell cycle control, mitosis and meiosis
[Y]	0	0.00	Nuclear structure
[V]	116	2.03	Defense mechanisms
[T]	93	1.63	Signal transduction mechanisms
[M]	154	2.70	Cell wall/membrane biogenesis
[N]	15	0.26	Cell motility
[Z]	0	0.00	Cytoskeleton
[W]	4	0.07	Extracellular structures
[U]	24	0.42	Intracellular trafficking and secretion
[O]	120	2.10	Posttanslational modification, protein turnover,chaperones
[X]	22	0.39	Mobilome: prophages, transposons
[C]	298	5.22	Energy production and conversion
[G]	207	3.63	Carbohydrate transport and metabolism
[E]	230	4.03	Amino acid transport and metabolism
[F]	73	1.28	Nucleotide transport and metabolism
[H]	211	3.70	Coenzyme transport and metabolism
[I]	603	10.57	Lipid transport and metabolism
[P]	223	3.91	Inorganic ion transport and metabolism
[Q]	388	6.80	Secondary metabolites biosynthesis, transport and catabolism
[R]	503	8.82	General function prediction only
[S]	181	3.17	Function unknown

The genome of AFP-003^T^ has the genetic potential to produce secondary metabolites, with 39 genes found to be associated with polyketide synthases and non-ribosomal peptide syntases. *M*. *ahvazicum* genome exhibits an average nucleotide identity of 86% with *M*. *genavense*, 82% with *M*. *simiae*, 81% with *M*. *interjectum*, 72% with *M*. *triplex*, 69% with *M*. *parascrofulaceum* and 68% with *M*. *sherrisii* (Tables 5, 6). In silico DNA-DNA hybridization analysis yielded 36.45% ± 3.46% with *M*. *triplex*, 32.55% ± 3.46% with *M*. *genavense*, 26% ± 3.39% with *M*. *sherresii*, 25.8% ± 3.39% with *M*. *simiae*, 24.7% ± 3.39% with *M*. *interjectum* and 24.2% ± 3.39 with *M*. *parascrofulaceum*. Ori-Finder^6^ was used to predict the origin of replication in the genome of strain AFP-003^T^. We found three OriC regions separated by the dnaA gene and located in scaffold 1 (218, 312 and 391 bp) (Supplementary File 1). The three predicted OriC region showed no homology sequence with those of the DoriC database7^7^. Contigs have been deposited (EBI accession number: FXEG02000000). Annotated genome is available at https://www.ebi.ac.uk/ena/data/view/PRJEB20293.

**Table 5:** Numbers of ortholog genes between genomes (upper right), average percentage similarity of nucleotides corresponding to orthologs between genomes (lower left) and number of ORFs per genome (bold); in selected *M*. *simiae* complex species including *M*. *ahvazicum* strain AFP-003^T^.

	*M*. *genavense*	*M*. *ahvazicum*	*M*. *interjectum*	*M*. *simiae*	*M*. *triplex*	*M*. *sherrisii*	*M*. *parascrofulaceum*
*M*. *genavense*	**5375**	3011	2417	2727	3036	2857	2646
*M*. *ahvazicum*	0.86	**5758**	3286	3585	3913	3672	3569
*M*. *interjectum*	0.81	0.81	**5953**	3002	3154	3008	3137
*M*. *simiae*	0.82	0.82	0.80	**5533**	3557	3502	3222
*M*. *triplex*	0.75	0.72	0.69	0.69	**5988**	3647	3404
*M*. *sherrisii*	0.68	0.68	0.67	0.71	0.68	**5020**	3299
*M*. *parascrofulaceum*	0.6	0.69	0.70	0.68	0.73	0.68	**6456**

**Table 6:** Comparison of *M*. *ahvazicum* AFP-003^T^ with related mycobacteria species using GGDC, formula 2 (DDH estimates based on identities/HSP length.

Species	DDH with *M*. *ahvazicum* AFP003^T^ (%)
*M*. *triplex*	36.45 ± 3.46
*M*. *genavense*	32.55 ± 3.46
*M*. *sherresii*	26 ± 3.39
*M*. *simiae*	25.8 ± 3.39
*M*. *interjectum*	24.7 ± 3.39
*M*. *parascrofulaceum*	24.2 ± 3.39

**Figure 5:**
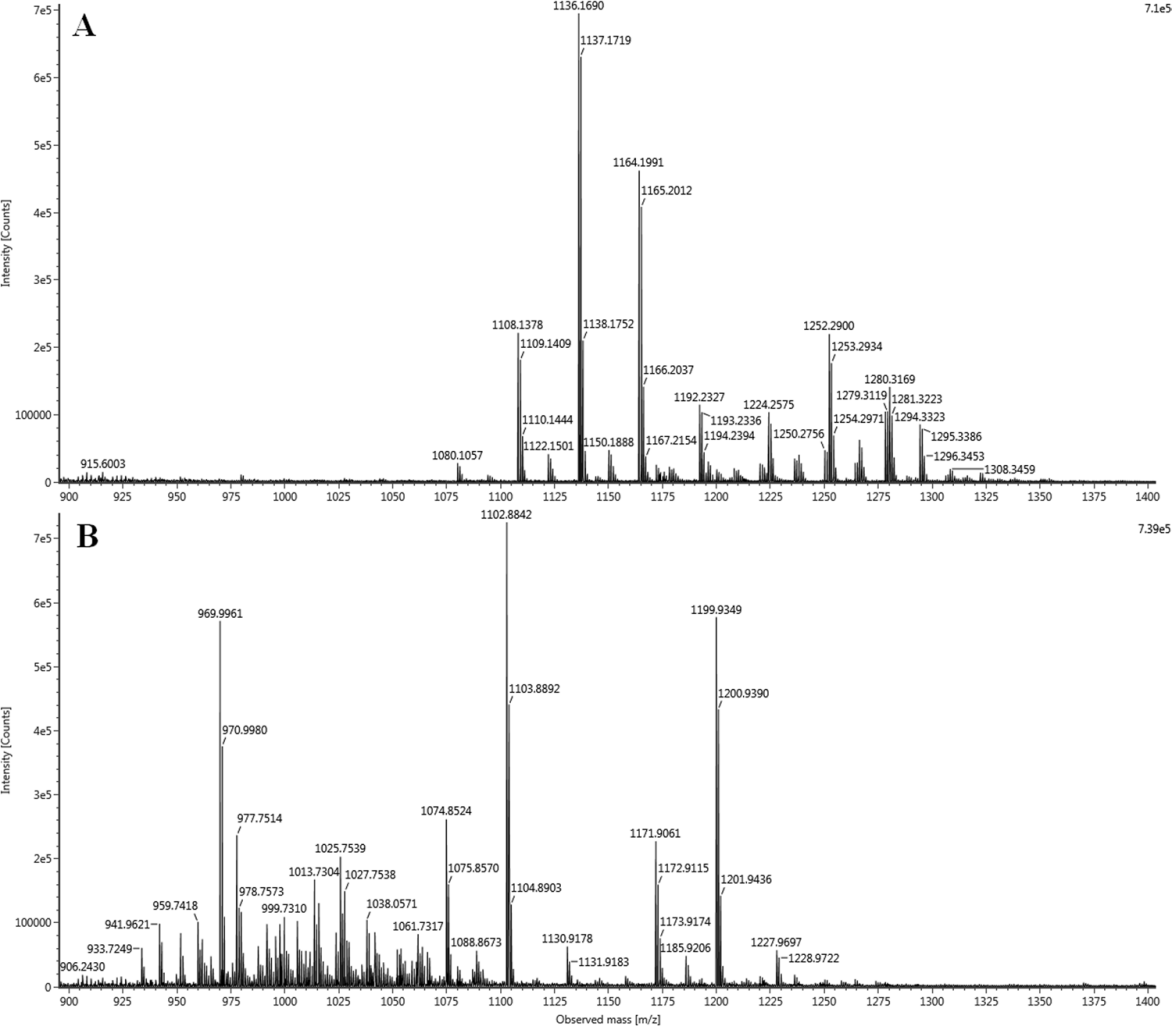
ESI-MS spectra of the [M − H]^−^ mycolic acid ions. (**A**) *Mycobacterium tuberculosis* H37Rv (control), (**B**) *Mycobacterium ahvazicum* AFP-003^T^.

To better describe AFP003^T^, the mycolic acids were identified. The mass spectrometry analysis of *Mycobacterium tuberculosis* H37Rv strain (used as a positive control) showed the previously described mycolic acid pattern^8,9^, including α- (C_74-84_), *methoxy-* (C_80-90_) and *keto-* (C_80-89_) forms. Strain AFP-003^T^ showed two known mycolic acids subclasses, α- (C71-74) and α′- (C64-68) forms, representing 15% of relative intensity defining an original mycolic acid profile (Table 7, Fig. 5).

**Table 7:** Identified mycolic acids for strains AFP-003^T^ and *Mycobacterium tuberculosis* H37Rv (control).

Mycolic acid subclass	Formula	Calculated [M − H]^−^	*Mycobacterium ahvazicum* AFP-003^T^			*Mycobacterium tuberculosis* H37Rv		
			Measured [M − H]^−^	Error (ppm)	%^a^	Measured [M − H]^−^	Error (ppm)	%^a^
α-	C_71_H_138_O_3_	1038.05732	1038.06117	3.7	1.5			
	C_72_H_140_O_3_	1052.07297	1052.07398	1.0	0.9			
	C_73_H_142_O_3_	1066.08862	1066.08942	0.8	0.9			
	C_74_H_144_O_3_	1080.10427	1080.10151	−2.6	0.4	1080.10569	1.3	1.0
	C_75_H_146_O_3_	1094.11992				1094.11679	−2.9	0.4
	C_76_H_148_O_3_	1108.13557				1108.13778	2.0	7.8
	C_77_H_150_O_3_	1122.15122				1122.15009	−1.0	1.5
	C_78_H_152_O_3_	1136.16687				1136.16903	1.9	25.0
	C_79_H_154_O_3_	1150.18252				1150.18015	−2.1	1.7
	C_80_H_156_O_3_	1164.19817				1164.19906	0.8	17.0
	C_81_H_158_O_3_	1178.21382				1178.21126	−2.2	0.8
	C_82_H_160_O_3_	1192.22947				1192.22827	−1.0	4.4
	C_84_H_164_O_3_	1220.26077				1220.25784	−2.4	1.1
α′-	C_64_H_126_O_3_	941.96342	941.96209	−1.4	1.5			
	C_66_H_130_O_3_	969.99472	969.99218	−2.6	8.4			
	C_68_H_134_O_3_	998.02602	998.02758	1.6	0.8			
*Keto-*	C_80_H_156_O_4_	1180.19309				1180.19739	3.6	0.7
	C_82_H_160_O_4_	1208.22439						
	C_84_H_164_O_4_	1236.25569				1236.26005	3.5	1.3
	C_85_H_166_O_4_	1250.27134				1250.27109	−0.2	1.8
	C_86_H_168_O_4_	1264.28699				1264.28423	−2.2	1.2
	C_87_H_170_O_4_	1278.30264				1278.30276	0.1	4.1
	C_88_H_172_O_4_	1292.31829				1292.31631	−1.5	0.3
	C_89_H_174_O_4_	1306.33394				1306.33735	2.6	0.4
*Methoxy-*	C_80_H_158_O_4_	1182.20874				1182.21141	2.3	0.3
	C_81_H_160_O_4_	1196.22439				1196.22575	1.1	1.1
	C_82_H_162_O_4_	1210.24004				1210.23864	−1.2	0.7
	C_83_H_164_O_4_	1224.25569				1224.25752	1.5	3.9
	C_84_H_166_O_4_	1238.27134				1238.27213	0.6	1.5
	C_85_H_168_O_4_	1252.28699				1252.29003	2.4	8.3
	C_86_H_170_O_4_	1266.30264				1266.30089	−1.4	2.5
	C_87_H_172_O_4_	1280.31829				1280.31691	−1.1	5.7
	C_88_H_174_O_4_	1294.33394				1294.33233	−1.2	3.8
	C_89_H_176_O_4_	1308.34959				1308.35587	4.8	0.8
	C_90_H_178_O_4_	1322.36524				1322.36553	0.2	0.6

^a^Relative intensity was calculated from the sum of detected monoisotopic peaks.

The unique phenotypic, genetic and genomic characteristics of AFP-003^T^ strain all support the fact that it is representative of a hitherto undescribed species in the genus *Mycobacterium*. We named this new species *Mycobacterium ahvazicum* sp. nov., derived from the name Ahvaz, the city in the southwest of Iran where the strain AFP-003^T^ (=JCM 18430) was discovered; and strain AFP-003^T^ is the type strain of *M*. *ahvazicum*. The data here reported indicated that *M*. *ahvazicum* is another new species belonging to the large *M*. *simiae* complex in which 18 new species have been reported over the last fifty years. Interestingly, seven of these isolates have been isolated from sputum^4,5,10–14^, five from cervical lymph nodes^15–19^, one from blood^20^, one from rhesus macaques^21^, two from fishes^22,23^, one from water^24^ and one from an unknown human clinical source^25^ (Table 8).

**Table 8:** Synopsis of the *M*. *simiae* complex species characterized since 1965.

*Species*	Isolation source	Clinical presentation	Isolation year	Characterisation year	Isolation site	Growth	Ref
*M*. *simiae*	Rhesus macaques	ND	ND	1965	Hungary	SGM	(21)
*M*. *intermedium*	Sputum	Pulmonary disease	ND	1993	Germany	SGM	(13)
*M*. *interjectum*	lymph node	Chronic lymphadenitis	ND	1993	Germany	SGM	(18)
*M*. *genavense*	Blood, bone marrow, livers, spleens, intestines, lymph nodes	Fever and diarrhea and experienced weight loss.	ND	1993	Geneva	SGM	(20)
*M*. *triplex*	Lymph node	ND	ND	1996	USA	SGM	(19)
*M*. *lentiflavum*	sputum, gastric juice, urine	Spondylodiscitis	1991–1993	1996	USA	SGM	(14)
*M*. *heidelbergense*	Cervical lymph nodes	Lymphadenitis	ND	1997	Germany	SGM	(15)
*M*. *kubicae*	Respiratory specimen	ND	1994–1997	2000	USA	SGM	(10)
*M*. *palustre*	Water		1993	2002	Finland	SGM	(24)
*M*. *montefiorense*	Moray eels	Granulomatous skin disease	2001	2003	USA	SGM	(22)
*M*. *parmense*	Cervical lymph node	Local swelling of submandibular	1999	2004	Italia	SGM	(16)
*M*. *sherrisii*	Clinical specimen	ND	1975	2004	USA	SGM	(25)
*M*. *saskatchewanense*	Sputum and pleural fluid	Bronchiectasis	2000	2004	Canada	SGM	(11)
*M*. *parascrofulaceum*	Sputum and bronchoscopy specimens	Symptoms of TB except for a dry cough	2002	2004	Canada	SGM	(12)
*M*. *florentinum*	Cervical lymph node	Lymphadenopathy	1993	2005	Italia	SGM	(17)
*M*. *stomatepiae*	Spleen tissue of *Stomatepia mariae*	Granulomatous lesions in spleen	ND	2008	London, UK	SGM	(23)
*M*. *europaeum*	Sputum (Human)	Cavitary pneumopathy	1995	2011	Italy, Florence	SGM	(5)
*M*. *paraense*	Sputum	Pulmonary symptoms	ND	2015	Brazil	SGM	(4)
*M*. *ahvazicum*	Sputum	Chronic Pulmonary disease	2009	2017	Iran	SGM	This work

SGM: Slowly Growing Mycobacteria, ND: No Data.

The discovery of *M*. *ahvazicum* is one more example illustrating that digging for mycobacteria in previously under-explored territories would reveal new species, as previously illustrated by our recent report of *Mycobacterium massilipolyniensis* in one remote island of the French Polynesian territories^26^.

## Methods

### Phenotypic characterization

Biochemical tests were carried out using standard methods^27^ and the minimal inhibitory concentration (MIC) of the major antimycobacterial agents was determined using the broth microdilution method^28^.

### Biolog Phenotype microarray

The ability of AFP-003^T^ to metabolize 71 different carbon substrates and resist to 23 inhibitory chemicals was tested using Gen III Microplates Biolog® Phenotype MicroArray (Biolog Inc)^29^. AFP-003^T^ was cultured at 37 °C on Middlebrook 7H10 agar medium supplemented with 10% (v/v) oleic acid/albumin/dextrose/catalase (OADC) (Becton Dickinson, Sparks, MD, USA) for 2 weeks. Colonies were gently taken with the wet swab off the agar plate culture and then rubbed against the wall of a dry glass tube. The cells were then suspended in IF-B (Biolog inoculating fluid recommended for strongly reducing and capsule producing bacteria, including Mycobacteria) and adjusted to 90% transmittance using a turbidimeter (Biolog Inc). Two plates (duplicate) were then inoculated and incubated in the OmniLog PM System (Biolog Inc.) at 37 °C for three days. The results were obtained as area under the curve (AUC) by Biolog’s parametric software.

### Transmission Electron Microscopy

The size of the microorganisms was determined by transmission electron microscopy (Morgani 268D; Philips, Eindhoven, The Netherlands) after negative staining at an operating voltage of 60 kV.

### Extraction and analysis of mycolic acids

AFP-003^T^ and *Mycobacterium tuberculosis* H37Rv (used a positive control) were cultured on Middlebrook 7H10 agar medium supplemented with 10% 0ADC for three weeks. Mycolic acids were prepared as detailed previously with modifications^8,30^. At least six inoculation loops were collected from a culture plate and transferred into 2 mL of potassium hydroxide 9 M. Mycolic acids were hydrolyzed at 100 °C during 2 hours. Free mycolic acids were then extracted with 2 mL of chloroform at low pH by adding 3 mL of 6 N hydrochloric acid. The organic phase was collected and dried at 40 °C under a stream of nitrogen. Free mycolic acids were then dissolved in 100 µL of a methanol-chloroform mixture (50:50, v/v) and subjected to electrospray-mass spectrometry analysis after a 2000 fold dilution in methanol. Samples were analyzed in the Sensitivity Negative ionization mode using a Vion IMS QTof high resolution mass spectrometer (Waters, Guyancourt, France). Samples were infused at 10 µL/min after fluidics wash with a chloroform/methanol solution (50:50) and monitored from 500 to 2000 m/z during 2 minutes. Ionization parameters were set as follow: capillary voltage 2.5 kV, cone voltage 50 V, source and desolvation temperatures 120/650 °C. Mass calibration was adjusted automatically during analysis using a Leucine Enkephalin solution at 50 pg/µL (554.2620 m/z). Mass spectra between 900 and 1400 m/z were used for subsequent data interpretation. Mycolic acids were described according to previously detailed structures^31^.

### MALDI-TOF-MS

Using a sterile 200 µL tip, a small portion of a colony was picked on a Middlebrook 7H10 solid-medium and applied directly on a ground-steel MALDI target plate. Then, one µL of a matrix solution (saturated α-cyano-4- hydroxycinnamic acid in 50% acetonitrile and 2.5% trifluoroacetic acid) (Bruker Daltonics) was used to over-lay the sample. After 5 minutes-drying at room temperature, the plate was loaded into the Microflex LT (Bruker Daltonics) mass spectrometer. Spectra were recorded following the parameters as previously described^32^. All signals with resolution ≥400 were automatically acquired using AutoXecute acquisition control in flexControl software version 3.0 and the identifications were obtained by MALDI Biotyper software version 3.0 with the Mycobacteria Library v2.0 database (version December, 2015).

### Phylogenetic analysis

Phylogenetic and molecular evolutionary analyses based on the 16S rRNA gene sequence were inferred using the maximum likelihood method implemented on MEGA7^33^, with the complete deletion option, based on the Tamura-Nei model for nucleotide sequences. Initial trees for the heuristic search were obtained automatically by applying the neighbor-joining and BIONJ algorithms to a matrix of pairwise distances estimated using the maximum composite likelihood (MCL) approach. Statistical support for internal branches of the trees was evaluated by bootstrapping with 1000 iterations.

### Genome sequencing

Total DNA of strain AFP-003^T^ was extracted in two steps: A mechanical treatment was first performed by acid-washed (G4649-500g Sigma) glass beads using a FastPrep BIO 101 instrument (Qbiogene, Strasbourg, France) at maximum speed (6.5 m/sec) for 90 s. Then after a 2-hour lysozyme incubation at 37 °C, DNA was extracted on the EZ1 biorobot (Qiagen) with EZ1 DNA tissues kit. The elution volume was of 50 µL. gDNA was quantified by a Qubit assay with the high sensitivity kit (Life technologies, Carlsbad, CA, USA) to 32.5 ng/µL. Genomic DNA was sequenced on the MiSeq Technology (Illumina Inc, San Diego, CA, USA) with the two applications: paired end and mate pair. Both strategies were barcoded to be mixed respectively with 11 other genomic projects prepared according to the Nextera XT 166 DNA sample prep kit (Illumina) and with 11 others projects according to the Nextera Mate 8 Pair sample prep kit (Illumina). To prepare the paired-end library, 1ng of gDNA was fragmented and amplified by limited PCR (12 cycles), introducing dual-index barcodes and sequencing adapters. After purification on AMPure XP beads (Beckman Coulter Inc, Fullerton, CA, USA), the libraries were normalized and pooled for sequencing on the MiSeq. Automated cluster generation and paired-end sequencing with dual indexed 2 × 250-bp reads were performed in a 9-hour run. Total information of 9.0 Gb was obtained from a 1,019 k/mm^2^ cluster density with a cluster passing quality control filters of 90.2% (17,374,744 passed filtered reads). Within this run, the index representation for AFP-003^T^ was determined to be of 8.20%. The 1,424,260 paired end reads were trimmed and filtered according to the read qualities. The mate pair library was prepared with 1.5 µg of genomic DNA using the Nextera mate pair Illumina guide. The genomic DNA sample was simultaneously fragmented and tagged with a mate pair junction adapter. The profile of the fragmentation was validated on an Agilent 2100 BioAnalyzer (Agilent Technologies Inc, Santa Clara, CA, USA) with a DNA 7500 labchip. The optimal size of obtained fragments was of 5.043 kb. No size selection was performed and 544 ng of tagmented fragments were circularized. The circularized DNA was mechanically sheared to small fragments with optima on a bi modal curve at 421 and 881 bp on the Covaris device S2 in T6 tubes (Covaris, Woburn, MA, USA). The library profile was visualized on a High Sensitivity Bioanalyzer LabChip (Agilent Technologies Inc, Santa Clara, CA, USA) and the final concentration library was measured at 16.97 nmol/L. The libraries were normalized at 2 nM, pooled with 11 other projects, denatured and diluted at 15 pM. Automated cluster generation and 2 × 250-bp sequencing run were performed in a 39-hour run. This library was loaded on two different flow cells. For each run, global information of 5.3 and 7.2 Gb was obtained respectively from a 559 and 765 K/mm2 cluster density with a cluster passing quality control filters of 96.3 and 94.7% (10,450,000 and 14,162,000 passed filter clustersfor each sequencing run). Within these runs, the index representation for AFP-003^T^ was determined to be of 8.51 and 7.62%. The 888,760 and 1,079,096 paired-end reads. The three runs leaded to a total of 3,392,116 paired-end reads which were filtered according to the read qualities. The reads were assembled using the SPAdes software (http://bioinf.spbau.ru/spades)^34^. Contigs obtained were combined by use of SSPACE^35^ assisted by manual finishing and GapFiller^36^. Open reading frames (ORFs) were predicted using Prodigal^37^ with default parameters. The predicted ORFs were excluded if they spanned a sequencing gap region (containing N). The predicted bacterial protein sequences were searched against the GenBank database and the Clusters of Orthologous Groups (COGs) database using BLASTP (E value 1e-03, coverage 0.7 and 30% identity). If no hit was found, it searched against the NR database using BLASTP with an E value of 1e-03, coverage 0.7 and 30% identity. The tRNAs and rRNAs were predicted using the tRNA Scan-SE and RNAmmer tools, respectively^38,39^. SignalP and TMHMM were used to foresee the signal peptides and the number of transmembrane helices, respectively^40,41^. For each selected genome, complete genome sequence, proteome genome sequence and Orfeome genome sequence were retrieved from the FTP site of National Center for Biotechnology Information (NCBI). All proteomes were analyzed using proteinOrtho^42^. An annotation of the entire proteome was performed to define the distribution of functional classes of predicted genes per cluster of orthologous groups of proteins (using the same method as for the genome annotation). The origin of replication was predicted using OriFinder^5,6^ (http://tubic.tju.edu.cn/Ori-Finder/) and homology with other OriC regions was searched using blast algorithm in DoriC database7 (http://tubic.tju.edu.cn/doric/). The *M*. *ahvazicum* strain AFP-003^T^ genome was further incorporated into in silico DNA-DNA hybridization (DDH)^43^ with reference genomes selected based on 16S rRNA gene proximity; and DDH values were estimated using the GGDC version 2.0 online tool^44^. For AFP-003^T^ genome comparison, we used the following species: of *M*. *parascrofulaceum*, *M*. *triplex*, *M*. *interjectum*, *M*. *genavense*, *M*. *sherrisii* and *M*. *simiae*.

## Electronic supplementary material

Supplementary Information
