# Supplementary material for: Mycobacterium ahvazicum sp. nov., the nineteenth species of the Mycobacterium simiae complex
Source: Sci Rep. 2018 Mar 7;8:4138. doi: 10.1038/s41598-018-22526-z (PMC5841666; doi:10.1038/s41598-018-22526-z)
Supplement: Supplementary file 1 — Supplementary Information [file 41598_2018_22526_MOESM1_ESM.pdf]

***Mycobacterium ahvazicum* sp. nov., the nineteenth species of the *Mycobacterium simiae* complex**

Running title: *Mycobacterium ahvazicum* sp. nov.

Amar Bouam[1,2]*, Parvin Heidarieh[3]*, Abodolrazagh Hashemi Shahraki[4], Fazel Pourahmad[5], Mehdi Mirsaeidi[6], Mohamad Hashemzadeh[7], Emeline Baptiste[1,2], Nicholas Armstrong[1,2], Anthony Levasseur[1,2], Catherine Robert[1,8] and Michel Drancourt[1,2]

SUPPLEMENTARY INFORMATION

Supplementary File 1. Information of Mycobacterium ahvazicum strain AFP003^{T} OriC region.

DnaA box distribution [DnaA box distribution]

OriC length 278 nt

OriC AT content 0.4245

The number of DnaA box 3

The location of oriC region

20137..20414 nt

The location of dnaA gene -

The extremes of GC disparity 353 nt (minimum), 2938724 nt (maximum)

The extremes of AT disparity 2916486 nt (minimum), 5940361 nt (maximum)

The extremes of RY disparity 56684 nt (minimum), 3195960 nt (maximum)

The extremes of MK disparity 2938761 nt (minimum), 353 nt (maximum)

Note Note that the DnaA box motif (ttgtccaca) was searched for with no more than two mismatches instead of E. coli perfect DnaA box (ttatccaca). [Gene list (zcurve1.02)]

Z-curves [Figure1] [Figure2]

OriC Sequence The DnaA boxes identified in the below sequence are capitalized and also marked in bold, if any.

cacctttttcgaccgtgcttgcaatagtcggtcagtacctacttgTGCGGAAAAcaactc

gggctcgggctgatcggctcgaacggaaaatgggtcaccccatctccccgcgtgataatg

cactcgctcacgcgggaggtgcgttttcaatcttgggtcgcagacctcgcgtctccctcc

tgccctgggaggtgcgatgtcgccggataattcgcggtttagtcgtctaaaggcgctact

cgagtcgaagCTGTCGACATgaccaggttctcggcaga

DnaA box distribution [DnaA box distribution]

OriC length 312 nt

OriC AT content 0.3590

The number of DnaA box 3

The location of oriC region

83512..83823 nt

The location of dnaA gene -

The extremes of GC disparity 353 nt (minimum), 2938724 nt (maximum)

The extremes of AT disparity 2916486 nt (minimum), 5940361 nt (maximum)

The extremes of RY disparity 56684 nt (minimum), 3195960 nt (maximum)

The extremes of MK disparity 2938761 nt (minimum), 353 nt (maximum)

Note Note that the DnaA box motif (ttgtccaca) was searched for with no more than two mismatches instead of E. coli perfect DnaA box (ttatccaca). [Gene list (zcurve1.02)]

Z-curves [Figure1] [Figure2]

OriC Sequence The DnaA boxes identified in the below sequence are capitalized and also marked in bold, if any.

tcgtaggcaagggtcggcgtggtctcggccggaTTGCCGACAcacgaagcgcccttacag

ctggtgacgaccccaccGGTGGACAGgtgcgccgactgctccggcgaaagggtctggcaa

tacacctcgtcgggcagaccgggttcgcgcttgtagttgacctcgcagctgatgttgcgc

gacggagacaaaaacgagcacagcgatgccgtgcagacggggTTGTCCGCGgccgccagg

ggcatgcttacgcgggagccgaggacgatggatccgattgtcgcgacgaccgcacgtttc

atgctgtcaaac

DnaA box distribution [DnaA box distribution]

OriC length 391 nt

OriC AT content 0.3811

The number of DnaA box 3

The location of oriC region

6058706..6059096 nt

The location of dnaA gene -

The extremes of GC disparity 353 nt (minimum), 2938724 nt (maximum)

The extremes of AT disparity 2916486 nt (minimum), 5940361 nt (maximum)

The extremes of RY disparity 56684 nt (minimum), 3195960 nt (maximum)

The extremes of MK disparity 2938761 nt (minimum), 353 nt (maximum)

Note Note that the DnaA box motif (ttgtccaca) was searched for with no more than two mismatches instead of E. coli perfect DnaA box (ttatccaca). [Gene list (zcurve1.02)]

Z-curves [Figure1] [Figure2]

OriC Sequence The DnaA boxes identified in the below sequence are capitalized and also marked in bold, if any.

agcgtcgaagacaaggcattggttgctgacctcgagttcggttgcagtaaaggaggtttc

gctgcggcgccggtggtcatcgtggtatgcgctgacacggaccgggtgccggagatctat

gcgccgccgtcgatttacccggcTGTGCAGAAtatgcttctggctgccgcagaccttggc

tacggttcgtgtTTGACCACCggcTTGACCACGtttggtgtcgatcaggtgcgggagctg

ttggaactgccgcagaatctgatcccgatggccgcagtctatgtcggtctgcccgcacgc

aagctctcaccgcctcgccgccgcccggccacatcgctgacatgccgcgagcggttcggt

acaccgtggtgaccagattggagagccgcta
